# Measuring the quality of nursing clinical placements and the development of the Placement Evaluation Tool (PET) in a mixed methods co-design project

**DOI:** 10.1186/s12912-020-00491-1

**Published:** 2020-10-29

**Authors:** Simon Cooper, Robyn Cant, Donna Waters, Elise Luders, Amanda Henderson, Georgina Willetts, Marion Tower, Kerry Reid-Searl, Colleen Ryan, Kerry Hood

**Affiliations:** 1 School of Health, Federation University Australia, Room 2W-249, Gippsland Campus, Churchill, VIC Australia; 2 Federation University Australia, Churchill, VIC Australia; 3 The University of Sydney Susan Wakil School of Nursing and Midwifery, M02C - 88 Mallett Street - Building C, Sydney, Australia; 4 School of Nursing and Healthcare Professions, Federation University, Gippsland Campus, Churchill, VIC Australia; 5 School of Nursing Midwifery and Paramedicine, University of the Sunshine Coast, Locked Bag 4, Maroochydore, DC, QLD 4558 Australia; 6 Department of Health Professions Faculty of Health, Arts and Design, Swinburne University, Hawthorn, VIC 3122 Australia; 7 School of Nursing, Midwifery & Social Work, University of Queensland, St Lucia, QLD 4072 Australia; 8 School of Nursing, Midwifery and Social Sciences, CQUniversity, Rockhampton, Australia; 9 School of Nursing, Midwifery and Social Sciences, CQUniversity Australia, Noosa Campus, Noosaville, QLD Australia; 10 Nursing at Holmesglen Institute, Melbourne, Australia

**Keywords:** Clinical placement, Clinical practicum, Education, Quality improvement, Students, Nursing

## Abstract

**Background:**

The quality of nursing clinical placements has been found to vary. Placement evaluation tools for nursing students are available but lack contemporary reviews of clinical settings. Therefore, the aim of this study was to develop a feasible, valid and reliable clinical placement evaluation tool applicable to nursing student placements in Australia.

**Methods:**

An exploratory mixed methods co-design project. Phase 1 included a literature review; expert rating of potential question items and Nominal Group Technique meetings with a range of stakeholders for item development. Phase 2 included on-line pilot testing of the Placement Evaluation Tool (PET) with 1263 nursing students, across all year levels at six Australian Universities and one further education college in 2019–20, to confirm validity, reliability and feasibility.

**Results:**

The PET included 19-items (rated on a 5-point agreement scale) and one global satisfaction rating (a 10-point scale). Placements were generally positively rated. The total scale score (19 items) revealed a median student rating of 81 points from a maximum of 95 and a median global satisfaction rating of 9/10. Criterion validity was confirmed by item correlation: Intra-class Correlation Co-efficient ICC = .709; scale total to global score *r* = .722; and items to total score ranging from .609 to .832. Strong concurrent validity was demonstrated with the Clinical Learning Environment and Supervision Scale (*r* = .834). Internal reliability was identified and confirmed in two subscale factors: Clinical Environment (Cronbach’s alpha = .94) and Learning Support (alpha = .96). Based on the short time taken to complete the survey (median 3.5 min) and students’ comments, the tool was deemed applicable and feasible.

**Conclusions:**

The PET was found to be valid, reliable and feasible. Use of the tool as a quality assurance measure is likely to improve education and practice in clinical environments. Further international evaluation of the instrument is required to fully determine its psychometric properties.

## Background

Nursing education programs across the world incorporate clinical placement experiences to assist learners to assimilate theory and practice. Approaches to placement quality assessment vary from ‘in-house’ reviews by education and clinical providers to the use of published student, educator and organisational survey instruments [1]. Internationally, the quality of clinical placements is known to vary with reported positive [2], ambivalent [3] and negative experiences [4]. Clinical learning environments are varied and complex with multidimensional social networks which makes evaluation complex.

In Australia, the Deans of Nursing and Midwifery (Australia and New Zealand) commissioned work to improve the quality of placements, which in the first instance required the development of a contemporary instrument to measure students’ placement experiences. As such the aim of this study was to develop a feasible, valid and reliable clinical placement evaluation tool applicable to nursing student placements in Australia.

*(NB: the use of the word ‘supervisor’ in this paper refers to the role of Registered Nurse mentor/ facilitator/ educator which, depending on the clinical placement model, may be a tertiary or organisational based position).*

Undergraduate nursing students are required to complete clinical placement hours as part of their educational preparation. Internationally these hours vary from 800 h in Australia, 1100–1500 in New Zealand, 2300 in the UK, and 2800 in South Africa [5]. It is accepted that exposure to quality ‘real world’ clinical placement is essential to ensure competence and appropriate development of professional identity; whilst the literature identifies that organisational, relational and individual factors influence the quality of placements [6].

Within organisations there is a need for a consistent approach between educational and industry sectors to ensure appropriate management of clinical placements [7]. Enabling a sense of belonging during placement ensures that students feel welcome [8] whilst the support of a clinical supervisor generates a positive learning environment.

Relations that are encouraging and supportive promote mutual respect, trust and open and honest communication [6]. Consistent and positive approaches from supervisors can overcome challenging clinical situations [9] whilst an awareness of students’ level of competence and learning requirements improve outcomes. Effective supervisors are well versed in the curriculum, clinical expectations and teaching practice whilst being motivated and approachable [7].

Individual students also harbour wide ranging interpretations of the clinical setting depending on their experience, resilience, and ‘life skills’, with the need to reduce vulnerability and create a positive learning culture [10]. Thus, preparation of nursing students for graduate practice requires engagement in the learning process and accountability for their learning. Frameworks that support active learning across educational and clinical settings and learning partnerships between supervisors and students are known to improve the quality of clinical placements [11].

With these considerations in mind it is imperative that rigorous evaluation instruments are available that measure the quality of placement experience, enabling improvements at placements sites and enhancing educational opportunities. There is therefore a climate of readiness for change and an essential need to develop a valid, reliable and feasible contemporary evaluation instrument that promotes national standards in clinical placement [12]. The following sections describe the development of the Placement Evaluation Tool (PET).

## Methods

An exploratory mixed methods project incorporating participatory co-design principals was planned to actively involve those who will become ‘users’ of the tool throughout the development process [13]. Such user-centric methods included individuals with lived experience of clinical placements (i.e. students, lecturers, supervisors, etc.) engaged as active design partners to generate ideas, prototype, gather feedback and make changes [14]. Incorporating these principals, the aim was to develop a deep understanding of clinical placements and relevant high utility assessment approaches. The project was undertaken and supported by a project team of 10 nursing academics in seven Australian tertiary educational institutions across three states. The project included a Phase 1 tool development stage, incorporating six key steps, and Phase 2 pilot testing.

### Ethical approval

Ethical approval for Phase 2 of the project (pilot testing) was obtained from the lead institution (Federation University Australia B19–070) with reciprocal approval from a further six institutions/pilot sites. Informed consent was required based on the participant information sheet provided at the start of the survey. No incentives, such as gifts, payments, or course credits were offered for participation.

#### Phase 1: tool development

##### Stage 1: literature review

A literature review was conducted to identify existing placement evaluation instruments. Ten original tools published between 1995 and 2015 were identified, incorporating a total of 303 rated items (e.g. [1, 9, 15–17]).

Overall there was a lack of contemporaneous language, international and cultural differences, grammatical and translation errors and outdated contexts. Further, from a feasibility perspective, most tools were considered too lengthy with the majority including over 30 items.

At this stage the project team decided not to include negatively worded items based on their tendency to cause confusion. Acquiescence was thought to be unlikely as participants would be rating personal clinical experiences [18]. Further, for feasibility, transferability and dissemination the tool was developed as a one page document, with generic questions that are applicable for clinical placements in Australia and with potential for future testing in other health professions and countries.

##### Stage 2: review of published items

Two researchers reviewed the identified items, removing duplications and non-applicable statements, leaving 190 items for consideration. An expert panel of six clinical academics (mean years of nurse registration - 32) rated the ‘Relevance’ and ‘Clarity’ of these items to produce an Item Content Validity Index (I-CVI) [19]. This enabled the exclusion, after discussion, of items that did not reach an acceptable level, i.e. an I-CVI of < 0.78. Approximately half the items were relevant and clear and were retained for further deliberation. Finally, several items from other broad generic training evaluation tools were selected e.g. Q4T [16] and H-PEPSS [15] with the intent of triangulating items with data generated in the Nominal Group meetings in the selection and adaption stage (described below in Stage 4).

##### Stage 3: nominal group meetings

The Nominal Group Technique (NGT) is designed to generate ideas, explore opinions and determine priorities [20], with previous use in, for example, diabetes education [21] and emergency care [22]. The Delphi Technique is an alternative consensus generating approach, however questionnaires are circulated anonymously, as opposed to face-to-face meetings in the Nominal Group Technique, enabling a greater exploration of the field of focus [20].

Two Nominal Group University based meetings were held, one in the State of Victorian and the second in the State of Queensland, Australia. The aim was to generate ‘fresh’ or ‘novel’ additional question items related to clinical placement quality from participants with first-hand experience. In order to comply with the co-design principals of the PET project we recruited a convenience sample from a range of stakeholders in each University region to attend one of the two three-hour meetings. Participants were recruited by a researcher at each site aiming to ensure adequate representation. In the Victorian group two 2nd year students, three 3rd year students, two graduate year nurses, one clinical placement coordinator and one clinical educator attended. In the Queensland group two 2nd year students, five 3rd year students, two clinical placement coordinators and two nursing academics attended. Total attendees for the two groups was therefore 20.

The Nominal Group Technique is described in detail elsewhere [23] but in summary the process included:

1. An introduction to the project aim and the NGT process.
2. Silent/individual generation of potential survey items on cue cards.
3. Round robin listing of items with discussion.
4. Group discussion and clarification of items.
5. Ranking of items.
6. Review and discussion regarding final listings.

By the end of each meeting a set of high priority evaluation statements was identified based on individual participants’ ranking. Ranking was achieved by accepting only high priority items prioritized by at least three participants. Fifty-six items in total were carried over to the next stage.

##### Stage 4 - selection and adaption of items

The principal researcher (SC) performed an independent primary analyses of items, followed by a five-hour meeting with three additional clinical researchers. Their clinical experience ranged from 27 to 37 years (mean 32). Potential items from the above stages were selected, adapted and thematisised using a paper based tabletop approach. The principal researcher’s initial development was then used as a reference point/check aiming for consensus. Individual items were listed under key themes e.g. supervision, the culture of the clinical environment, learning outcomes. A priori specification of items to Kirkpatrick’s evaluation model [24] - Level 1 (Reaction to the experience/clinical environment), Level 2 (Learning outcomes) and Level 3 (Behavioural change/practice impact) was also performed at this point. Items were then selected and wording was adjusted if necessary, generating a 20 item questionnaire.

A five point Likert scale was selected with a scale ranging from [1] ‘strongly disagree’ to [5] ‘strongly agree’. An even numbered scale (forced choice) was not selected as participants were likely to require a mid-point response i.e. ‘neither agree or disagree’. Further, a five point scale enabled a direct concurrent validity comparison with another validated tool - the Clinical Learning Environment and Supervision Scale [17] (described below). A 20th item was included, as an overall satisfaction rating, with a response scale of 1 (very dissatisfied) to 10 (extremely satisfied).

##### Stage 5 – tool review (educators and students)

The draft tool was then circulated to 10 clinical educators from the Australian states of Queensland, New South Wales, and Victoria and to 12 nursing students from Queensland and Victoria, in order to calculate the I-CVI prior to final selection. The expected I-CVI of >.78 was exceeded for relevance and clarity in all but three educator rated items, which were resolved with minor changes to wording. For example item 6 was originally worded “Patient safety was integral to the work of the unit(s)” and was reworded to “Patient safety was fundamental to the work of the unit(s)”, to ensure greater clarity.

##### Stage 6 - deans of nursing review

A final review was provided by 37 Deans of Nursing and Midwifery (Australia and New Zealand) at a meeting in Queensland (July 2019) where minor wording changes to the demographic section were adopted.

#### Phase 2: pilot testing and validation

##### Stage 1 - pilot testing

The tool was pilot tested through an on-line survey at six Australian universities and one Technical and Further Education (TAFE) institution where Bachelor of Nursing degree students were enrolled (i.e. excluding Enrolled Nurse trainees). These sites were selected as they were led by a project team member who was also the Dean of School or their representative. One site ran a two year graduate entry Masters program whose students were excluded and a double degree nursing/midwifery four year program, where students were surveyed only after a nursing placement.

Purposive population sampling aimed to include all 1st, 2nd, 3rd and 4th year nursing students who had completed a clinical placement in 2nd Semester (July 2019 to-February 2020). Invitations to complete the PET were distributed by a clinical administrator at each site, who provided the survey access link and distributed e-mail reminders. In this pilot testing phase, participants and their review sites were not identifiable. Participants were asked to rate their ‘most recent’ clinical placement only.

The survey was uploaded to Qualtrics survey software (Qualtrics, Provo, UT, USA) enabling anonymized student responses. Three academics tested the survey for accuracy, flow, and correct response options. Access to the Participant Information Statement was enabled and consent requested via a response tick-box. Seven questions regarding demographics were included e.g. age group, year of study course, placement category. This was followed by the 20-item PET and two open ended questions relating to students’ placement experience and suggestions for improving the PET. Access to the survey was enabled via smart phones and computers. The survey remained open between July 2019 and February 2020 whilst students were completing their placements. Finally, 62 students were approached at one university in order to measure the concurrent validity of the PET against the Clinical Learning Environment and Supervision Scale. The test-retest reliability of the PET, with the same test seven days later, was reported by 22 students from two universities.

##### Stage 2

In this final stage the aim was to confirm the validity, reliability and feasibility of the PET using applicable statistical and descriptive analyses. Outcomes are described in the results section below.

### Data analysis

Survey data downloaded from the Internet were analysed using IBM SPSS vs 26 [25]. Descriptive and summary statistics (means, standard deviations) were used to describe categorical data whilst between group associations were explored using inferential statistics (*t* tests, ANOVA). Pearson’s product moment correlational analysis of item-to-total ratings and item-to global-scores was conducted. The Intra-class Correlation Co-efficient (2-way random-effects model) [26] was used to examine inter-item correlation. P = < 0.05 was regarded as significant. The internal consistency reliability was computed using Cronbach’s alpha.

A Principle Component Analysis was conducted to identify scale items that grouped together in a linear pattern of correlations to form component factors, using the method of Pallant [27]. The sample exceeded the recommendation of at least 10 participants for each variable. The factorability of data was confirmed by Bartletts’s test of sphericity (<.0.5) of p = <.001 and the Kaiser-Meyer-Olkin (KMO) measure of sampling adequacy (range 0–1, .6 minimum) of .97. The high KMO of .97 indicates a compact range of correlations with data appropriate for factor analysis ([28] p.877). An eigenvalue > 1 was applied to extract the number of factors and a Scree plot showed two components. The correlation matrix was based on correlations above .3. Assisted by the large sample, the variables loaded strongly, as described below.

Prior to analyses the normality of the total scale score was confirmed by the Kolmogorov-Smirnov statistic (0.148, df 1263, p = < 0.001) and Shapiro-Wilk Test (0.875, df 1263, p = < 0.001). Although positive skewness was noted with scores clustered towards higher values (Skewness: 1.327, Kurtosis: 1.934), these data were within the acceptable normal distribution range [27].

## Results

The validity and reliability of the PET was based on responses from 1263 pre-registration nursing students who completed the survey (see Table 1). The response rate was estimated at 20.2% (1263/6265). The sample comprised students enrolled in the first to fourth years of a nursing degree. Participants represented three Australian States but the majority were in Queensland (45.9%) or Victoria (44.3%). Nearly all were female (89.9%); most were in the second year of their course (42.9%) and the most common age group was 20–25 years (31.9%). The majority were responding about their experiences of clinical placement in an acute health services setting (54.5%) followed by Mental Health (19.4%) or Aged Care (17.7%).

**Table 1:** Characteristics of nursing student sample (*n* = 1263)

VARIABLE	CATEGORY	NUMBER (%)
**Gender**	Female	1133 (89.8)
	Male	127 (10.1)
	Other	1 (0.1)
**Age group**	19 or younger	156 (12.4)
	20–25	402 (31.9)
	26–30	144 (11.4)
	31–35	152 (12.1)
	36–40	141 (11.2)
	41–45	121 (9.6)
	46–50	86 (6.8)
	51 or older	59 (4.7)
**State of enrolment**	New South Wales	123 (9.7)
	Queensland	580 (45.9)
	Victoria	560 (44.3)
**Degree type**	Single degree	1222 (96.8)
	Double degree	41 (3.2)
**Year of degree**	First year	321 (25.4)
	Second year	542 (42.9)
	Third year	385 (30.5)
	Fourth year	15 (1.2)
**Last placement setting**	Acute hospital	688 (54.5)
	Mental Health	245 (19.4)
	Aged Care	223 (17.7)
	Rehabilitation service	63 (5.0)
	Primary care/ community	38 (3.0)
	Other	6 (0.5)
**Placement duration (days)**	First year	1–80 (mode = 10)
	Second year	2–80 (mode = 15)
	Third year	10–80 (mode = 30)
	Fourth year	14–60 (mode = 30, 55)

### Summary of participant ratings

Placements were generally positively rated. The total scale score (19 items) revealed a median student rating of 81 points from a maximum of 95 and a mean of 78.3% [95% CI: 77.4–79.2; SD 16.0]. Table 2 lists the means responses for each item.

**Table 2:** Summary statistics for nursing students’ response to the prototype PET (n = 1263)

Scale item	Mean	SD
1. I was fully orientated to the clinical area	4.06	1.10
2. Staff were willing to work with students	4.11	1.04
3. Staff were positive role models	4.02	1.03
4. Staff were ethical and professional	4.10	0.96
5. Staff demonstrated respect and empathy towards patients/clients	4.18	0.90
6. Patient safety was fundamental to the work of the unit(s)	4.33	0.85
7. I felt valued during this placement	3.88	1.18
8. I felt safe in the clinical environment (e.g. physically, emotionally culturally)	4.20	0.95
9. This placement was a good learning environment	4.16	1.14
10. My supervisor(s) helped me identify my learning objectives/needs	4.03	1.12
11. I was adequately supervised in the clinical environment	4.17	1.01
12. I received regular and constructive feedback	3.94	1.15
13. I was supported to work within my scope of practice	4.20	1.01
14. My supervisor(s) understood how to assess my clinical abilities	4.06	1.20
15. I had opportunities to enhance my skills and knowledge	4.13	1.11
16. I had opportunities to interact and learn with the multi-disciplinary team	4.09	1.08
17. I achieved my learning objectives	4.17	0.99
18. I have gained the skills and knowledge to further my practice	4.22	0.94
19. I anticipate being able to apply my learning from this placement	4.26	0.93
Overall		
20. Overall, I was satisfied with this placement experience.	8.74	1.77

Although every scale item had a response range of between 1 and 5, there was positive skewness towards higher ratings; 17 of 19 items were rated above a mean of 4.0 of five points. The highest rated item was [6]. ‘Patient safety was fundamental to the work of the unit(s)’, with a mean of 4.33, followed by item [19]. ‘I anticipate being able to apply my learning from this placement’ (M = 4.26). The lowest rated items were [7] ‘I felt valued during this placement’ (M = 3.88) and ‘I received regular and constructive feedback’ (M = 3.94). Such responses indicate areas for future exploration.

Item 20 overall satisfaction with the placement experience was rated as high (median 9 of 10) with 377 (29.8%) participants being ‘extremely satisfied’ (10 out of 10) and an additional 686 (54.3%) rating between 6 and 9. A total of 38 students (3.0%) were ‘very dissatisfied’ and a further 101 (8.0%) were dissatisfied and rated the experience between 2 and 4 points. The open-ended comments provided by participants may help to deconstruct these issues in future.

The new instrument was able to differentiate perceptions of placement quality when total scores were classified across the three States. Mean total scale scores were significantly higher for Victorian students (M = 80.68) than for New South Wales (M = 78.55) and Queensland (M = 76.01) students (F = 12.395, df2, p = < 0.001). This difference was also reflected in the Global Satisfaction rating (F = 9.360, df2, p = < 0.001) with Victorian students reporting significantly higher mean global satisfaction (M = 8.98), Queensland (M = 8.56) and New South Wales (M = 8.50).

### Validity and reliability outcomes

The first objective in developing a measurement instrument is to demonstrate its validity - the degree to which it measures what it is intended to measure. This can be established using several statistical approaches including assessment of face/content validity, and construct validity [14]. The second main requirement is to test the scale reliability; the extent to which measurements are free from error and can be replicated, generally measured with correlational tests. Below, we describe the findings and present a summary in Table 3.

**Table 3:** Validity and reliability of the Placement Evaluation Tool (PET) (19-items)

Variable	Sample	Result	Significance (***p***-value)	Outcome
**Construct validity**				
Content validity:	12 students	.82		Valid: >.78
I-CVI: Stage 5	10 educators	.95		Valid: >.78
Concurrent validity:				
Correlation with CLES scale	62 students	.834	0.01	Valid: highly significant
Criterion validity:				
(a) Item to Total score	1263	.606 to .832	< 0.001	Valid
(b) Scale vs Global score	1263	.722	0.01	Valid >.7: highly significant.
(c) Inter-Item correlation *(ICC: Intraclass Correlation Coefficient).*	1263	.709 (CI: .692–.727)	< 0.001	Valid- .5–.75 = good correlation
**Reliability**				
Internal consistency reliability (Cronbach’s alpha)				
(1). Clinical Environment	1263	.94	N/A	Reliable (>.70)
(2). Learning Support	1263	.96	N/A	Reliable (>.70)
Scale: Test and retest (*Wilcoxon signed rank test)*	22 students	z = −1.705	.088	Acceptable non-significant difference at retest

Adequate construct validity was demonstrated by content validity measures, concurrent and criterion related validity all of which reached or exceeded expected values. In development stages the expertise of educators and students was used as a filtering mechanism to assure face validity and usability with acceptable outcomes from the I-CVI.

Concurrent validity with a volunteer sample of second year nursing students (*n* = 62) in Victoria was measured using both the PET and the Clinical Learning Environment and Supervision Scale [17, 29]. Correlation was high *r =* .834 supporting the notion that the PET had high concurrent validity.

Criterion validity was measured via inter-item correlations, item-to-total score and correlation of the scale total score with the independent ‘global’ score. The 19 items were moderately to strongly correlated. The Intraclass Correlation Coefficient (random effects model) of .709 for single measures showed non-significant differences across the 19 scale items (p = < 0.001) - classified as a ‘good’ correlation [26]. The corrected item-to-total correlation for the scale ranged from .606 to .832 and Friedman’s Chi-square confirmed consistency (p = < 0.001). There was no redundant outlier item with a low correlation. The total scale score was also strongly correlated with the independent global score (*r* = .722, *p* = 0.01) (two-tailed).

Test-retest with a sample of 22 nursing students from two states confirmed the stability of scores over time, indicated by non-significant difference at retest after one week (Z = − 1.705, *p* = 0.088).

### Factor analysis

PCA was conducted to ascertain how the pattern of correlated items was able to describe experience. Analysis using Varimax rotation yielded a two-factor solution that explained 73.3% of the variance. The first factor had an eigenvalue of 12.66 and explained 66.63% of the variance; the second, an eigenvalue of 1.27, explaining 6.66% of the variance (see Table 4).

**Table 4:** Principal component analysis outcome: rotated matrix (n = 1263)

Item	Component	
	1	2
1. I was fully orientated to the clinical area	**.451**	.452
2. Staff were willing to work with students	**.745**	.432
3. Staff were positive role models	**.807**	,422
4. Staff were ethical and professional	**.844**	.329
5. Staff demonstrated respect and empathy towards patients/clients	**.825**	<.300
6. Patient safety was fundamental to the work of the unit(s)	**.760**	.352
7. I felt valued during this placement	**.684**	.531
8. I felt safe within the clinical environment (e.g. physically, emotionally and culturally)	**.717**	.440
9. This placement was a good learning environment	.566	**.664**
10. My supervisor(s) helped me identify my learning objectives/needs	<.300	**.784**
11. I was adequately supervised in the clinical environment	.454	**.705**
12. I received regular and constructive feedback	.379	**.770**
13. I was supported to work within my scope of practice	.456	**.736**
14. My supervisor(s) understood how to assess my clinical abilities	.311	**.792**
15. I had opportunities to enhance my skills and knowledge	.402	**.797**
16. I had opportunities to interact and learn with the multi-disciplinary team	.419	**.709**
17. I achieved my learning objectives	.342	**.829**
18. I have gained the skills and knowledge to further my practice	.404	**.785**
19. I anticipate being able to apply my learning from this placement	.404	**.784**

Extraction Method: Principal Component Analysis. Rotation Method: Varimax with Kaiser Normalization

a Rotation converged in 3 iterations

The two factors that emerged were clinically meaningful: items number 1–8 formed one component that was labelled Factor 1 ‘Clinical Environment’. Items 9–19 formed a second component which was labelled Factor 2 ‘Learning Support’. Both subscales were found reliable: (1) ICC = .937 (CI .931–.942), *p* = < 0.001; (2) ICC = .964 (CI .961–.967), *p* = < 0.001.

In addition to test-retest reliability the Cronbach alpha statistic is a measure of the internal reliability/consistency with a range of 0–1 and an expected standard ≥ .7. The alpha reliability of the PET scales was: (1) Clinical Environment .94 (8 items); (2) Learning Support .96 (11 items). While these data appear high, inspection of the item-total correlation matrix for each scale revealed tightly clustered correlations with no downward influence on the overall alpha if a single item was removed [30].

### Translational impact: Kirkpatrick’s four level model of evaluation

Good practice in educational evaluation has been described as incorporating four levels of evaluation [24]. Table 5 illustrates how items in the PET scale address the first three levels: Reaction, Learning and Behaviour. Level 4 Results - patient impact was not applicable in this instance.

**Table 5:** Translation of PET items to Kirkpatrick’s levels of evaluation

Kirkpatrick’s Level	PET Factor	PET Scale Items
**LEVEL 1:** Reaction to experience	**Clinical Environment**	(1) I was fully orientated to the clinical area (2) Staff were willing to work with students (3) Staff were positive role models (4) Staff were ethical and professional (5) Staff demonstrated respect and empathy towards patients/clients (6) Patient safety was fundamental to the work of the unit(s) (7) I felt valued during this placement (8) I felt safe in the clinical environment (e.g. physically, emotionally and culturally)
**LEVEL 2:** Learning	**Learning Support**	(9) This placement was a good learning environment (10) My supervisor(s) helped me identify my learning objectives/needs (11) I was adequately supervised in the clinical environment (12) I received regular and constructive feedback (13) I was supported to work within my scope of practice (14) My supervisor(s) understood how to assess my clinical abilities (15) I had opportunities to enhance my skills and knowledge (16) I had opportunities to interact and learn with the multi-disciplinary team (17) I achieved my learning objectives
**LEVEL 3** Behaviour change	**Learning Support**	(18) I have gained the skills and knowledge to further my practice (19) I anticipate being able to apply my learning from this placement
**LEVEL 4** Patient impact	*Not applicable*	

### Students’ open text comments

Respondents were asked how the PET could be improved. The few responses received indicate that the overall tool was ‘good’, relevant and clear. Students’ comments about their personal placement experiences were numerous and diverse and will be described in a later report.

### Feasibility

The tool was planned as a short online survey in order to increase participant acceptability, however there was a degree of attrition with 83% of 1524 who accessed the survey completing all items. Most who exited withdrew at or before the first mandatory scale item (14%).

In relation to completion time, noting that some participants may have left the survey open to return at a later date, 16 outliers (duration > 1 h) were removed identifying a median completion time of 3.5 min (SD 4.5) (range 1.1 mins to 44.6 mins).

## Discussion

There is international evidence that clinical placement experiences vary considerably (e.g. 4). Organisational management, supervisory relations and student expectations need to be considered in order to adequately prepare nursing students for safe graduate practice [6]. With these concerns in mind we aimed to produce a feasible, valid and reliable clinical placement evaluation tool that would enable students to rate the clinical and educational environment and their learning experience, generating a national profile of placement experiences and quality.

The final PET includes 20 plain English items measuring two key factors – ‘Clinical Environment’ and ‘Learning support’ and three Kirkpatrick evaluation domains - participant reactions to the experience/clinical environment, self-reported learning outcomes and behavioural change/practice impact. Whilst reactions to an experience and self-reported outcomes are frequently measured in surveys, measures of practice impact are less frequently covered [31]. However, hard measures of observed practice performance, as opposed to self-reports, would further enhance reviews of placement activity. As shown in Table 3, the tool exhibited statistically valid and reliable properties in all respects tested, for example reliability was established with a Cronbach alpha of .94 for the Clinical Environment scale and an alpha of .96 for the Learning Support scale.

The two key factors identified reflect the importance of a welcoming atmosphere and educational support, as expressed in many other published instruments (e.g. 29). In the current study, despite the high global satisfaction rate (median 9/10), 11% of respondents were dissatisfied, with comments relating to negative staff attitudes and the working environment. This finding is of concern and confirms the need for a quality assessment tool and regular placement reviews.

The final participant open access PET is listed in Additional file 1. Nineteen items are rated on a scale of 1 to 5 and the final global rating from 1 to 10, with potential scores ranging from 20 to 105. A summed score of the first 19 items and the overall global rating are likely to be useful in feedback processes. No quality assessment ‘cut score’ i.e. acceptable or unacceptable placements have been set, as institutions should consider individual placement evaluations from multiple students with a combination of evaluation approaches. In this pilot trial of the PET institutions/students were not identified, but for quality improvement future sites must be identified to enable feedback and action.

Future research will aim to produce a placement evaluation tool that is applicable across health disciplines in the developed world. As such this primary development of the PET is limited as it focusses on one discipline –nursing, three States in one country – Australia and in the English language only. Future iterations will therefore be required including a national Australian nursing trial, testing and development for other health disciplines and rigorous translations (forward- backward) into additional languages. Additionally, larger sample sizes are necessary to be sure of the test-retest reliability. Broader limitations of such tools must also be considered as the PET is an individual self-rating of experience with the need to take into account additional stakeholders reviews e.g. educators and hard outcome measures such as practice observation, student retention, employment offers etc.

In summary, widespread use of a tool such as the PET, perhaps as a suite of assessment tools within a national registry of clinical placements, is likely to have an impact on both educational and clinical outcomes through applicable quality improvement programs that ensure the right education, in the right place and at the right time.

## Conclusion

In a survey of 1263 nursing students in Australia the PET was found to be valid, reliable and feasible across a range of measures. Use of the tool as a quality improvement measure is likely to improve educational and clinical environments in Australia. Further evaluation of the instrument is required to fully determine its psychometric properties. Future work with the PET will include a national nursing survey across all Australian States and Territories, international nursing surveys and additional health discipline trials.

## Supplementary information

**Additional file 1.** Finalised Placement Evaluation Tool (PET).

## Data Availability

The datasets generated and/or analysed during the current study are not publicly available due to confidentiality but anonymised sets may be available from the corresponding author on reasonable request.

## Abbreviations

NGT: Nominal Group Technique
PET: Placement Evaluation Tool
PCA: Principal component analysis
