# Supplementary material for: Measuring the quality of nursing clinical placements and the development of the Placement Evaluation Tool (PET) in a mixed methods co-design project
Source: BMC Nurs. 2020 Oct 29;19:101. doi: 10.1186/s12912-020-00491-1 (PMC7594450; doi:10.1186/s12912-020-00491-1)
Supplement: Supplementary file 1 — Additional file 1. Finalised Placement Evaluation Tool (PET). [file 12912_2020_491_MOESM1_ESM.docx]

**The Placement Evaluation Tool (PET)**
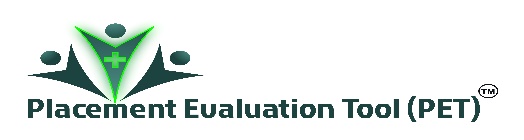


**Appendix A**

| *The student placement evaluation tool is found overleaf. In addition to this, institutions may wish to*  *collect the following data. Please edit as required.*  **Citation:** Cooper S, Cant R, Waters D, Luders E, Henderson A, Willetts G, Tower M, Reid-Searl K, Ryan C, & Hood K, 2019.  **Funding:** Council of Deans of Nursing and Midwifery (Australia and New Zealand). |
| --- |
| **Introduction** |

This survey has been designed to enable you, as a student, to evaluate your clinical placement. Please rate your experience and return this form to your university placement co-ordinator. This feedback will enable quality improvements with benefits to educators and students.

| **Your details** |
| --- |

1. **Student name ……………………………………… Student ID: …………………………….**
2. **In which University/TAFE are you enrolled?**

**………………………………………………………………………………………………………..**

1. **In which State or Territory?**

Australian Capital Territory ^1^

New South Wales ^2^

Northern Territory ^3^

Queensland ^4^

South Australia ^5^

Tasmania ^6^

Victoria ^7^

Western Australia ^8^

1. **In which year of a degree are you enrolled?** (if a combination, list highest year)

First year  Second year  Third year  Fourth year 

1. **In which specialty/field was your most recent clinical placement?**

Acute hospital ^1^ Rehabilitation service ^2^ Aged care ^3^ Primary care ^4^

Mental Health ^5^ Other ^6^ (Please name) …………………………………………………

1. **What is the name of your placement health service?**

…………………………………………….……………………………………………………………

1. **When did you attend placement (dd/mm/yyyy)?**

First day: ..…./ ....../…....

1. **My primary clinical supervisor was a:**

Registered Nurse ^1^ Clinical Educator ^2^ University Educator ^3^

**The Placement Evaluation Tool (PET)**

*Use this scale to rate question items about your placement experience**:*

| **Strongly disagree** | **Disagree** | **Neither agree or disagree** | **Agree** | **Strongly agree** |
| --- | --- | --- | --- | --- |
| 1 | 2 | 3 | 4 | 5 |

***Please circle the number that best matches your opinion***

|  | ***Rating*** |
| --- | --- |

| 1. I was fully orientated to the clinical area | 1 | 2 | 3 | 4 | 5 |
| --- | --- | --- | --- | --- | --- |
| 1. Staff were willing to work with students | 1 | 2 | 3 | 4 | 5 |
| 1. Staff were positive role models | 1 | 2 | 3 | 4 | 5 |
| 1. Staff were ethical and professional | 1 | 2 | 3 | 4 | 5 |
| 1. Staff demonstrated respect and empathy towards patients/clients | 1 | 2 | 3 | 4 | 5 |
| 1. Patient safety was fundamental to the work of the unit(s) | 1 | 2 | 3 | 4 | 5 |
| 1. I felt valued during this placement | 1 | 2 | 3 | 4 | 5 |
| 1. I felt safe in the clinical environment *(e.g. physically, emotionally, culturally)* | 1 | 2 | 3 | 4 | 5 |
| 1. This placement was a good learning environment | 1 | 2 | 3 | 4 | 5 |
| 1. My supervisor(s) helped me identify my learning objectives/needs | 1 | 2 | 3 | 4 | 5 |
| 1. I was adequately supervised in the clinical environment | 1 | 2 | 3 | 4 | 5 |
| 1. I received regular and constructive feedback | 1 | 2 | 3 | 4 | 5 |
| 1. I was supported to work within my scope of practice | 1 | 2 | 3 | 4 | 5 |
| 1. My supervisor(s) understood how to assess my clinical abilities | 1 | 2 | 3 | 4 | 5 |
| 1. I had opportunities to enhance my skills and knowledge | 1 | 2 | 3 | 4 | 5 |
| 1. I had opportunities to interact and learn with the multi-disciplinary team | 1 | 2 | 3 | 4 | 5 |
| 1. I achieved my learning objectives | 1 | 2 | 3 | 4 | 5 |
| 1. I have gained the skills and knowledge to further my practice | 1 | 2 | 3 | 4 | 5 |
| 1. I anticipate being able to apply my learning from this placement | 1 | 2 | 3 | 4 | 5 |

| 1. **Overall,** I was satisfied with this placement experience.   (Decide your overall rating on a scale of 1-10)  *(1 being very dissatisfied, 10 being extremely satisfied)* | 1 | 2 | 3 | 4 | 5 | 6 | 7 | 8 | 9 | 10 |
| --- | --- | --- | --- | --- | --- | --- | --- | --- | --- | --- |

**Please feel free to add additional comments about your placement experience:**

--------------------------------------------------------------------------------------------------------------------------------------------------------------

--------------------------------------------------------------------------------------------------------------------------------------------------------------

--------------------------------------------------------------------------------------------------------------------------------------------------------------
